# SOX10-Mediated Regulation of Enteric Glial Phenotype in vitro and its Relevance for Neuroinflammatory Disorders

**DOI:** 10.1007/s12031-025-02321-y

**Published:** 2025-02-21

**Authors:** Madlen Kunke, Meike Kaehler, Sebastien Boni, Katja Schröder, Alicia Weier, Rittika Chunder, Stefanie Kuerten, Martina Böttner, Ingolf Cascorbi, Michel Neunlist, Thilo Wedel, Ralph Lucius, François Cossais

**Affiliations:** 1 Institute of Anatomy, Kiel University, Kiel, Germany; 2 Institute of Experimental and Clinical Pharmacology, University Hospital Schleswig-Holstein, Campus Kiel, Kiel, Germany; 3 Lentivec Platform, Université d’Angers, Angers, France; 4 Institute of Neuroanatomy, Medical Faculty, University of Bonn and University Hospital Bonn, Bonn, Germany; 5 Nantes University, Inserm, TENS, the Enteric Nervous System in Gut and Brain Diseases, IMAD, Nantes, France

**Keywords:** Enteric glial cells, Schwann cell, Sox10, PLP1, Nestin, Multiple sclerosis, Neuroinflammation

## Abstract

The transcription factor SOX10 is a key regulator of myelinated glial cell phenotype and function, with a known role in multiple sclerosis (MS). SOX10 is also expressed in enteric glial cells (EGC) within the gut, yet its regulatory functions in EGC remain poorly understood. This study aimed to identify SOX10 target genes that influence EGC phenotype and may have implications for MS. An EGC cell line was established for doxycycline-inducible SOX10 overexpression. Impact of SOX10 overexpression on EGC phenotype was assessed by genome-wide expression analysis and results were validated via RT-qPCR and western blot. Data were compared with SOX10 ChIP-seq and transcriptomic datasets from MS patients to identify pan-glial SOX10 target genes potentially linked to neuroinflammatory disorders. SOX10 overexpression was associated with ectopic upregulation of genes related to myelin regulation and glial differentiation, as evidenced by increased PLP1 expression at mRNA and protein levels. Comparison to ChIP-seq and MS datasets highlight SOX10 target genes, including *PLP1*, *RNF130*, *NES* and *APOD* potentially involved in central and peripheral manifestations of MS pathology. Our findings support a cell-specific regulation of EGC phenotype through SOX10 expression level and identify SOX10-regulated genes relevant to EGC function. This research advances the understanding of EGC diversity and provide information about glial cells targeting in neuroinflammatory disorders.

**Supplementary Information:**

The online version contains supplementary material available at 10.1007/s12031-025-02321-y.

## Introduction

The transcription factor SOX10 plays a pivotal function in the development and plasticity of myelinating glial cells. In particular, SOX10 regulates the differentiation and phenotype of oligodendrocytes and peripheral Schwann cells (Britsch et al. 2001) through the direct regulation of myelin gene expression. SOX10 has also been associated with alterations of the oligodendrocyte phenotype in relation with multiple sclerosis (MS) (Yeung et al. 2019).

SOX10 has been shown to promote the differentiation of human pluripotent stem cells toward an oligodendrocyte phenotype. Furthermore, *Sox10* overexpression is sufficient to induce transdifferentiation of astrocytes to an oligodendrocyte phenotype (Pozniak et al. 2010; García-León et al. 2018). Consequently, it has been proposed that SOX10-induced transdifferentiation toward an oligodendrocyte-phenotype may represent a strategy of interest toward the development of stem-cell based therapy in MS and neurodegenerative disorders (Mokhtarzadeh Khanghahi et al. 2018; Yavarpour‐Bali et al. 2020; Mozafari et al. 2020).

The enteric nervous system (ENS) plays a pivotal role in regulating main functions of the gastrointestinal tract. Besides its role in oligodendrocytes and Schwann cells, SOX10 plays an evolutionary conserved role in ENS development and Sox10 expression persists in enteric glial cells (EGC) after birth (Cossais et al. 2010, 2016). Whereas *Sox10* deletion leads to ENS developmental disorders, manifesting as Hirschsprung’s disease in humans, *Sox10* overexpression in enteric neural crest stem cells or enteric progenitors cells results in the maintenance of a progenitor state and inhibition of neuronal or glial differentiation (Kim et al. 2003; Bondurand et al. 2006).

Originally considered as mere supportive cells for enteric neurons, EGC have emerged as crucial contributors to the regulation of intestinal motility as well as to the maintenance of barrier functions, and the orchestration of neuro-immune interactions within the intricate intestinal microenvironment (Seguella and Gulbransen 2021; Progatzky and Pachnis 2022; Bubeck et al. 2023). Conversely, alterations in the phenotype and plasticity of EGC are emerging as pivotal factors in the pathophysiology of various diseases, including inflammatory bowel disease (IBD), and neuroinflammatory disorders, including MS (Neunlist et al. 2014; Wunsch et al. 2017; Chalazonitis and Rao 2018; Cossais et al. 2021). Nonetheless, little is known regarding the regulation of EGC phenotype under physiological or pathological conditions.

Recent research has illuminated the remarkable phenotypic diversity of EGC (Sharkey and Mawe 2023). Notably, six distinct EGC subtypes have been identified based on their localization and morphology (Seguella and Gulbransen 2021). These subtypes include EGC located within the myenteric and submucosal ganglia, EGC associated with interganglionic nerve fibers, and mucosal EGC. EGC express typical glial markers such as S100B, glial fibrillary acidic protein (GFAP), and SOX10. Whereas SOX10 and S100B serve as pan-EGC markers, the expression of GFAP appears to be more variable. In the last years, additional EGC markers have been identified, including proteolipid protein 1 (PLP1) (Rao et al. 2015). While transcriptomic evidence supports the distinct nature of EGC compared to central or to other peripheral glial cells, our understanding of the regulation of EGC phenotype remains limited (Boesmans et al. 2021; Elmentaite et al. 2021; Fawkner-Corbett et al. 2021). In particular, a definitive correlation between anatomical and molecular classifications of EGC remains an area of ongoing investigation, with the balance between plasticity and diversity yet to be fully elucidated (Valès et al. 2018).

In order to explore SOX10's role in EGC phenotypic plasticity, an EGC cell line overexpressing *Sox10* was developed. In order to identify genes relevant for MS cell therapy, SOX10-induced gene expression pattern in EGC was compared to transcriptomic datasets of central and peripheral glial cells as well as to transcriptomic profiles of MS patients. This approach further highlights SOX10 target genes potentially involved in the regulation of myelinating glial cells and EGC phenotype under physiological and pathological conditions (Esposito et al. 2013; Pochard et al. 2018; Wunsch et al. 2017).

## Material and Methods

### Cell Culture

The EGC line JUG2 derived from rat ENS primary culture was used for this study (Bach-Ngohou et al. 2010). JUG2 cells were cultured in cell specific growth medium (DMEM; Gibco; 41965–039) supplemented with 10% v/v fetal bovine serum (Pan-Biotech), 100 U/mL penicillin and 100 µg/mL streptomycin and incubated at 37 °C in a 5% CO_2_ and 95% air atmosphere. Cells were subcultured when they reached confluency by trypsinization. If not otherwise mentioned, SOX10 overexpression was induced by adding 1 µg/mL doxycycline to the cell growth medium and culturing cells for 48 h.

### Lentiviral Transduction of JUG2 Cells

To generate stable transgenic cell lines overexpressing SOX10, JUG2 cells were transduced with the following lentiviral vectors: Sox10 (TetO-FUW-Sox10, Addgene plasmid #45,843), transactivator protein (pLV EF1a TET3G, Addgene #184,379), GFP (pLV TREG EmGFP, Addgene #36,083). Vectors were produced by transient transfection of 293 T cells. A total of 5 × 10^6^ cells were seeded in a 10 cm dish in modified Dulbecco's culture medium supplemented with 10% fetal calf serum, penicillin (100 IU/mL) and streptomycin (100 µg/mL) in a 5% CO_2_ incubator. A total of 22.5 µg of plasmid DNA was used for transfection of one dish: 5 µg of envelope plasmid pMD.G, 7.5 µg of packaging plasmid psPax2, and 20 µg transfer vector plasmid. The precipitate was formed by adding the plasmids to a final volume of 227.5 µL water and 250 µL 2.5 M CaCl_2_, mixing well, then dropwise addition of 500 µL 2X HEPES buffered saline solution (281 mM NaCl, 100 mM HEPES, 1.5 mM, Na_2_HPO_4_ [pH 7.12]) while vortexing and immediately adding the precipitate to the cultures. The medium (10 mL) was replaced after 24 h. The conditioned medium was collected after a further 24 h, cleaned by low-speed centrifugation (400 × g for 5 min) and filtered through 0.45 mm PVDF (polyvinylidene difluoride) filters. One day before lentiviral transduction JUG2 cells were seeded in a density of 1 × 10^5^ cells in a 24-well plate. The following day JUG2 cells were incubated with a mix of 30 µL of EF1a TET3G and 50 µL of Sox10 (TetO-FUW-Sox10) or GFP (pLV TREG EmGFP) in presence of 5 µg/mL of puromycin under normal cell culture conditions. After 10 h transduction the media was changed to growth medium supplemented with 5 µg/mL of puromycin. After reaching confluency cells were transferred to 12-well plate followed by 6-well plate and further expansion. With each subcultivation we used 5 µg/mL puromycin in growth medium. Transduced JUG2 cells with TREG EmGFP instead of Sox10 served as fluorescent transduction control.

### Experimental Autoimmune Encephalomyelitis (EAE) Induction

Spinal cord and colonic tissues were retrieved from EAE-induced animals from a previous study (Weier et al. 2022). Briefly described, four C57BL/6 J females (Charles River; strain code 632), aged 9–12 weeks, were kept in pathogen-free conditions at the Franz-Penzoldt-Zentrum, University Hospital Erlangen. For EAE induction, MP4 (2 mg/mL, Alexion Pharmaceuticals) was mixed 1:1 with complete Freund’s adjuvant, containing paraffin oil (Sigma-Aldrich), mannide monooleate (Sigma-Aldrich), and *Mycobacterium tuberculosis* H37Ra (5 mg/mL, BD, Difco). 100 μL of the mixture was injected subcutaneously on each flank. Mice received 120 ng of pertussis toxin (Hooke Laboratories) diluted in 100 µL sterile PBS (Thermo Fisher) via intraperitoneal injection on the day of immunization and 24 h later. Efficiency of the induction has been previously evaluated (Weier et al. 2022). Untreated age-matched mice served as controls. All experiments were performed according to established protocols that were approved by the Government of Lower Franconia (“Regierung von Unterfranken”; license no. 55.2–2531.01–91/14) and the local Ethics Committee of Kiel University (V242-70,056/2015(91–7/15)) and in accordance with the 3R principles (Replacement, Reduction and Refinement) and the ARRIVE criteria.

### RNA Isolation and Real‑Time Quantitative PCR

Real-time quantitative PCR (RT-qPCR) was performed as previously published (Kneusels et al. 2021). In brief, total RNA extraction from JUG2 cell cultures or from mouse colon and spinal cord specimens was performed using Nucleozol (Macherey–Nagel) and the extracted RNA was stored at − 80 °C until further processing. Reverse transcription was performed on 1 μg total RNA using the Revert Aid reverse transcription kit (Thermo-Fisher Scientific) following manufacturer’s recommendations. Quantitative PCR (qPCR) was performed on 5 ng cDNA using the Evagreen Supermix (Solis biodyne) or the qPCR Master Mix Plus (Eurogentec) on an ABI Prism 7500 fast Real-Time PCR cycler (Life Technologies). The housekeeping gene *Rps6* was used for normalization as *Rps6* relative expression to *Gapdh* remained stable under the conditions used for the analyses (data not shown). Primer sequences are listed in Supplementary Table 1. Relative quantitation to control conditions was performed using the 2^–∆∆Ct^ method and normalization to control conditions.

### Microarray Analysis

Genome-wide expression analysis was performed on 100 ng total RNA JUG2 cells obtained from four independent experiments using Clariom S arrays (Thermo-Fisher Scientific) according to manufacturer’s recommendations and as previously published (Kneusels et al. 2021). Data were analyzed using the Transcriptome Analysis Console v4.0.3.14 and are available at the GEO database (GSE249205). Results were considered statistically significant for FDR-p-value < 0.01 and fold change ≥  ± 2. Genes matching afore mentioned filter criteria and induced only by doxycycline treatment were excluded for gene analysis.

### Gene Ontology Term Enrichment and Comparative Datasets Analysis

Gene Ontology (GO) term enrichment analysis was performed using the online tool Toppgene and using all genes differentially expressed more than tenfold after Sox10 induction together with a gene list derived from *Rattus Norvergicus*. Categories with a FDR-p-value < 0.05 were retained as “enriched”. Single-cell expression within the human ENS were obtained from a published dataset (Fawkner-Corbett et al. 2021) and analysed using the software R (version 4.1.0) running the Seurat library (version 4.0.3) (Hao et al. 2021) as previously described (Heimke et al. 2023). Genome-wide expression data for brain tissue of patients with multiple sclerosis was obtained from a published dataset (GSE38010) (Han et al. 2012). Expression observed in active plaques (GSM931814, GSM931815 and GSM931816) was compared to the expression in control tissues (GSM931812 and GSM931813) using the GEO2R online tool. DEG with fold change ≥ 2 and p-value < 0.05 were selected for further analysis. Analysis of overlapping DEG between given datasets was performed using the online tool Venn (https://bioinformatics.psb.ugent.be/webtools/Venn/).

### Immunofluorescence

JUG2 cells were grown on glass cover slips, fixed with 4% w/v paraformaldehyde (PFA)/PBS for 10 min, permeabilized with 0.5% v/v triton-100/PBS for 30 min and blocked with 5% v/v skim milk/PBS for 1 h. For detection of intermediate filaments, cells were incubated overnight at 4 °C with nestin antibody (dilution 1:100) followed by Alexa555-coupled (Invitrogen, dilution 1:500) antibody for 2 h at room temperature. Subsequently, cells were incubated overnight at 4 °C with SOX10 (dilution 1:100) as secondary primary antibody followed by Alexa488-coupled (Invitrogen, 1:500) antibody for 2 h at room temperature. Nuclei were counterstained with Hoechst 33342 (Sigma-Aldrich) for 5 min. Blank controls were performed by omitting primary antibodies. Image acquisition was performed using confocal microscopy with a “Facility Line” system (Abberior) based on an IX-83 inverted microscope (Olympus), running the Imspector 16.3.11308 software.

### Antibodies

The following antibodies were used: mouse anti-β-actin (sc-81178; Santa Cruz; Lot# E3012), rabbit anti-GFP (D5.1/#2956; Cell Signaling; Lot# 6), mouse anti-nestin (rat-401; Developmental Studies Hybridoma Bank), rabbit anti-PLP1 (NBP1-87781; Novus Biologicals; Lot# B115828) and rabbit anti-SOX10 (ILP3833; ImmunoLogic; Lot# 3947 V).

### Western Blot

Whole cell lysates were prepared according to standard protocols. Briefly, cells were washed once with PBS and mechanically collected by scraping in PBS, then centrifuged at 150 × g for 5 min. PBS was removed and cell pellets were resuspended in RIPA buffer including cOmplete Mini protease-inhibitor-cocktail, and PhosSTOP phosphatase inhibitor (both Roche Diagnostics GmbH, Mannheim). Lysates were incubated for 30 min at 4 °C, centrifuged at 17,500 × g at 4 °C for 45 min. Lysates were transferred into clean tubes and stored at − 20 °C. Protein concentration was measured using the Pierce™ BCA Protein Assay Kit (Thermo Fisher Scientific, Waltham, USA), and protein amounts were adjusted. Samples were denatured at 95 °C in Laemmli buffer for 5 min. Uncropped raw blots are shown in the supplementary data.

### Statistical Analysis

Statistical analysis was performed using the Prism software (Graphpad Version 5.04). Mann–Whitney U-test was used to perform comparison between two groups. One-way ANOVA (Kruskal–Wallis test) followed by Dunn´s post-test was performed to compare three groups or more. Data are provided as arithmetic means ± SEM with n representing the number of samples. Results were considered significant for *p ≤ 0.05, **p ≤ 0.01 and ***p ≤ 0.001.

## Results

### Establishment of SOX10 Overexpressing EGC Line

**Fig. 1:**
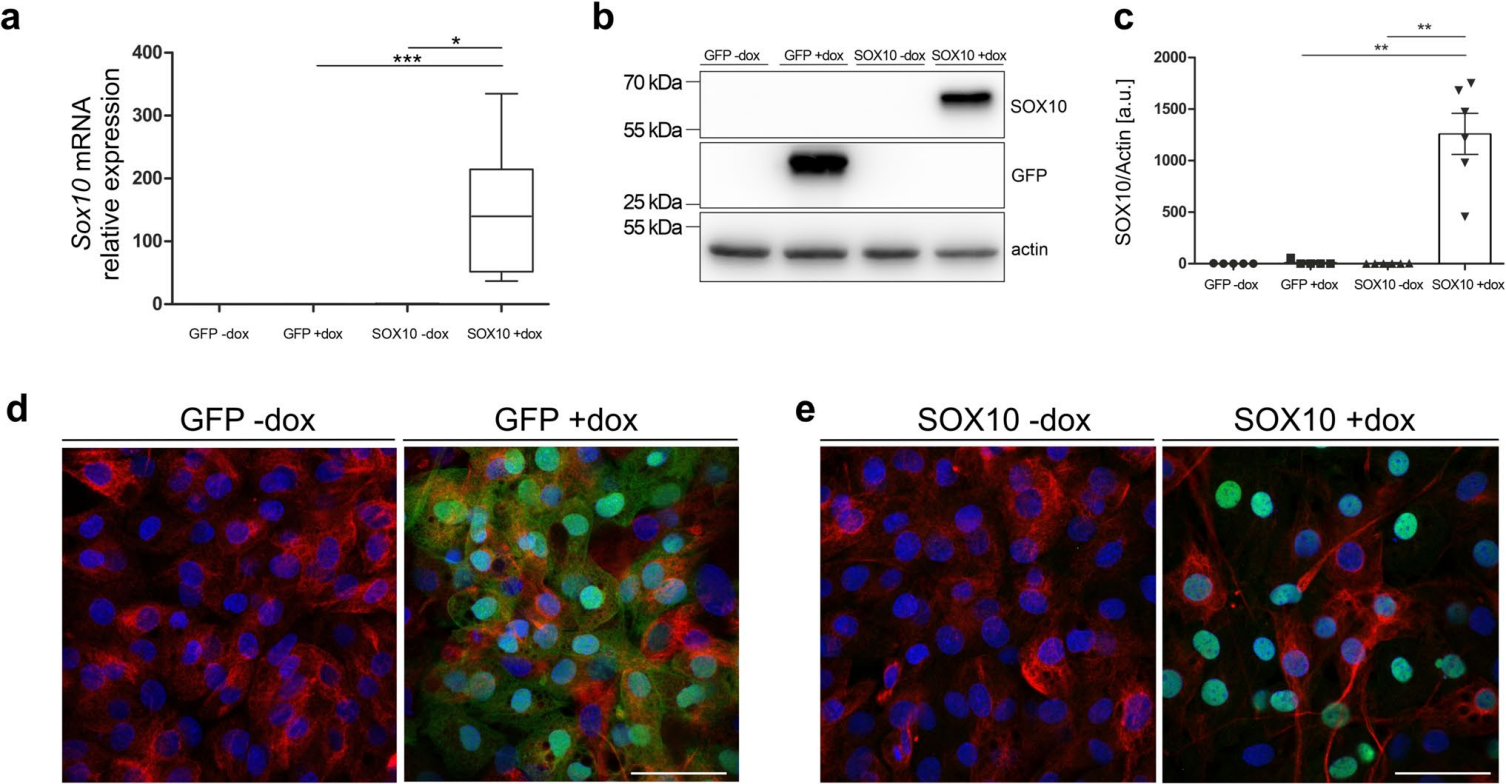
JUG2 cells after treatment with doxycycline (+ dox) showing overexpression of GFP (fluorescence control) and SOX10. **a** RT-qPCR. *n* = 3–8; *p ≤ 0.05, ***p ≤ 0.01 (Kruskal–Wallis test followed by Dunn's post-test). **b**, **c** Representative western blot of GFP, SOX10 and actin (loading control, b) and densitometrical analysis of SOX10 expression levels. *n* = 5–6; **p ≤ 0.01 (Kruskal–Wallis test followed by Dunn's post-test, c). **d**, **e** Representative confocal images of GFP (green, d) and of immunohistochemical stained SOX10 (green, e), nestin (red) and nuclei (blue). Scale bar = 50 µm

In order to investigate the putative role of SOX10 in the regulation of the EGC phenotype, we established a stably transduced EGC line with doxycycline-inducible overexpression of *Sox10*. In parallel, a control cell line overexpressing GFP in presence of doxycycline was similarly established. Induction of *Sox10* by treatment with doxycycline was first confirmed at mRNA level (Fig. 1a). *Sox10* mRNA levels were ~ 150-fold higher after treatment with doxycycline in comparison to untreated controls (SOX10 -dox) or to GFP control cell line (GFP + dox). Induction of GFP and SOX10 proteins by doxycycline in the respective cell lines was confirmed using western blot and immunofluorescence (Fig. 1b–e), validating the effectiveness of the experimental model.

### SOX10 Overexpression Induces Myelin Gene Expression in JUG2 Cells

**Fig. 2:**
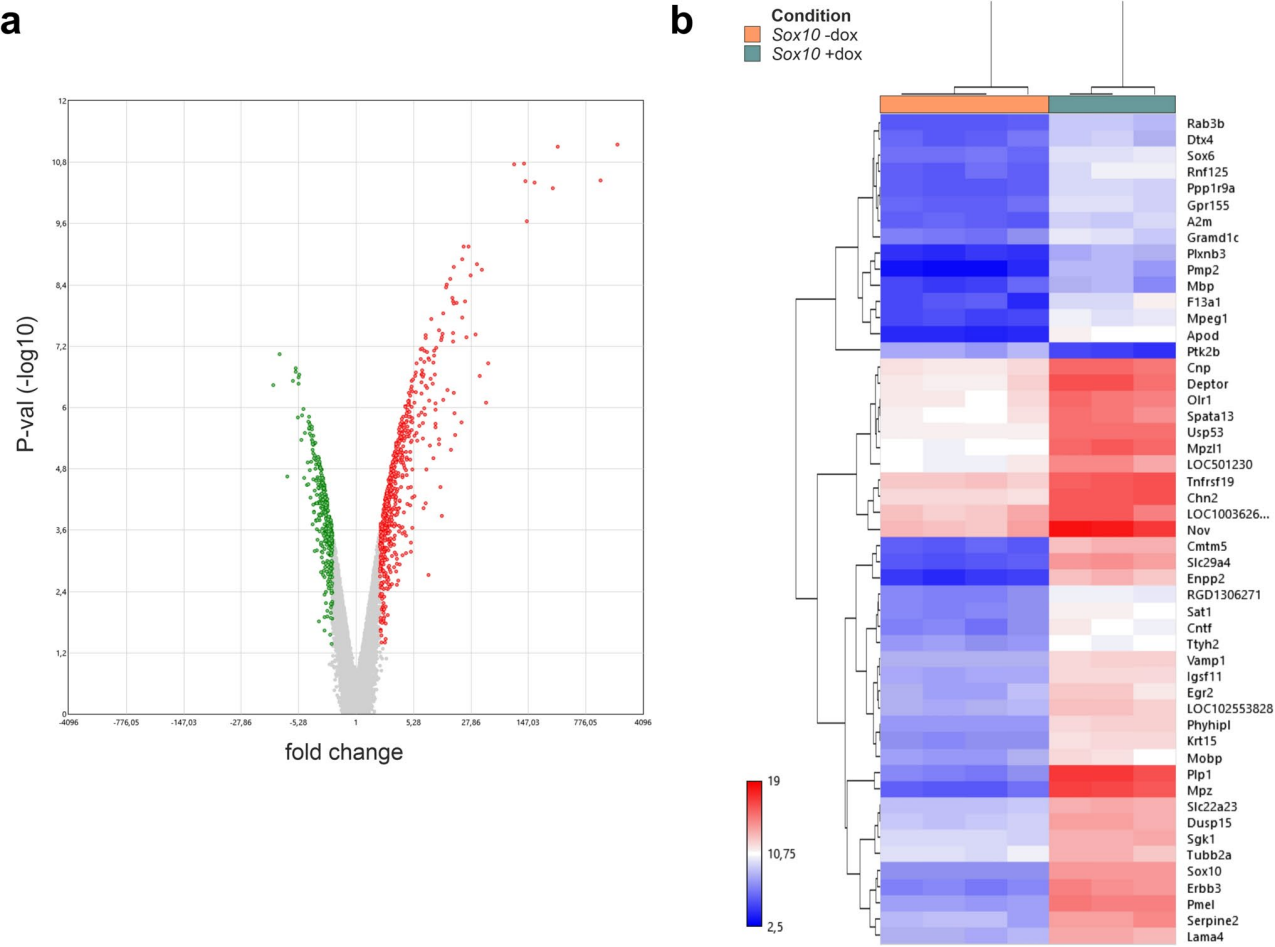
Impact of SOX10 overexpression on the transcriptome of JUG2 cells. **a** Volcano plots showing the differential gene expression between control and SOX10 overexpression. Red and green dots represent significant upregulated and downregulated genes, respectively (*n* = 3–4, fold change >  ± 2 and FDR-*p*-value < 0.05). **b** Hierarchical clustering of the main differentially expressed genes (fold change >  ± 10) between SOX10 -dox (control) and SOX10 overexpressing JUG2 cells

Subsequently, we conducted a comprehensive genome-wide expression analysis to assess the impact of SOX10 overexpression on the EGC phenotype. A total of 620 differentially expressed genes (DEG) showed increased expression levels and 356 DEG displayed down-regulation after doxycycline-mediated SOX10 induction (≥ ± two fold, Fig. 2, Supplementary Table 2).

In more detail, expression of genes closely associated with myelin regulatory pathways, including *Mpz, Plp1, Apod, Pmp2, Egr2 or Mobp*, were induced more than ± tenfold (Fig. 2b, Supplementary Table 2).

**Fig. 3:**
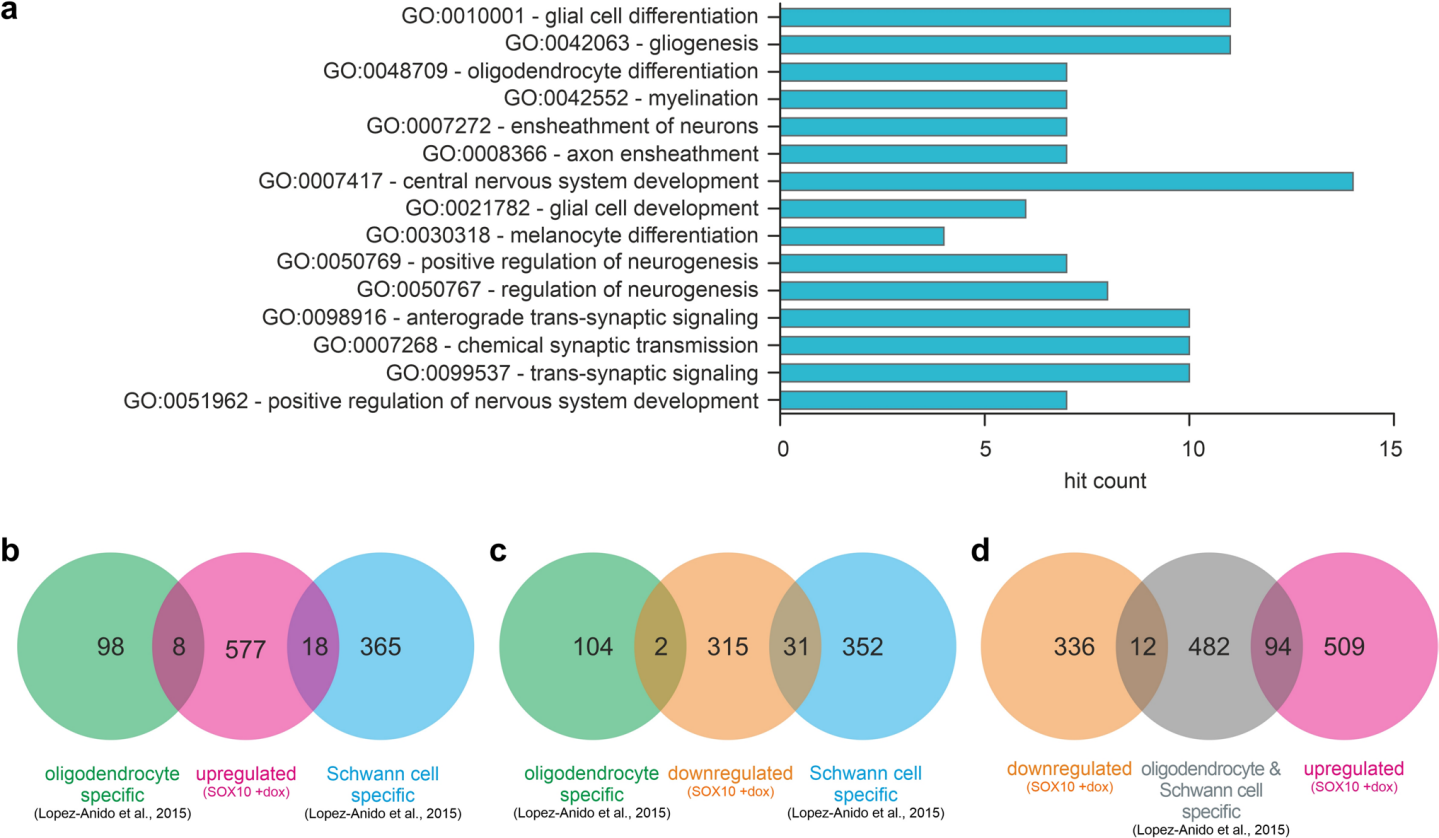
**a** Gene ontology (GO) analysis of SOX10 overexpressing JUG2 cells. JUG2 cells were incubated with doxycycline to induce SOX10 gene expression. Top 15 GO-molecular pathways fold enrichment of biological processes in doxycycline-treated SOX10 overexpressing JUG2 cells in comparison to control, based on differentially expressed genes (≥ 10-fold change). *FDR-p-value < 0.05. **b**, **c** Venn diagram representing the number of upregulated (b, pink) and downregulated (c, orange) DEG in SOX10-overexpressing JUG2 cells vs. oligodendrocyte-specific (green) and Schwann cell-specific (blue) SOX10 target genes, as determined by ChIP-seq analysis (Lopez-Anido et al. 2015). **d** Venn diagram representing the number of upregulated genes (pink) and downregulated (orange) DEG in SOX10-overexpressing JUG2 cells vs. SOX10 target genes common to oligodendrocytes and Schwann cells (grey), as determined by ChIP-seq analysis (Lopez-Anido et al. 2015)

We performed gene ontology (GO) enrichment analysis on the upregulated and downregulated DEG to assess the functional relevance of the induced altered gene expression panel. GO analysis confirmed the significant induction of cellular pathways related to glial cell differentiation and development (GO:0010001; GO:0021782; GO:0042063), myelination (GO:0042552), ensheathment of neurons and axons (GO:0007272; GO:0008366), but also central nervous system (CNS) development (GO:0007417, GO:0051962) and regulation of neurogenesis (GO:0050769, GO:0050767) (Fig. 3a, Supplementary Table 3). No significant alteration of gene expression was observed for the GFP overexpressing cell line after doxycycline induction in comparison to untreated control (data not shown). Therefore, further analyses were performed for the Sox10 overexpressing cell line only.

### Comparison of SOX10-Induced DEG with Oligodendrocyte and Schwann Cell-Specific ChIP-Seq Dataset Identifies Putative Pan-Glial SOX10 Target Genes

To gain more insight into the regulation of SOX10-induced DEG, we compared the set of DEG (≥ ± twofold change) from our analysis to a publicly available SOX10 ChIP-seq analysis dataset for Schwann cells and oligodendrocytes (Lopez-Anido et al. 2015) (Fig. 3b–d, Supplementary Tables 4–6). From the identified upregulated DEG, eight genes, including *Mobp*, were previously identified as oligodendrocyte-specific SOX10-target genes in the ChIP-seq analysis, whereas 18 genes, including *Cntf*, *Egr2* or *Nes*, were identified as Schwann cell-specific SOX10-target genes (Fig. 3b, Supplementary Table 4). Furthermore, two genes of 356 downregulated DEG were oligodendrocyte-specific whereas 31 genes were Schwann cell-specific (Fig. 3c, Supplementary Table 5). Besides this, we compared our DEG dataset (≥ ± 2-fold change) with SOX10-target genes identified in both oligodendrocytes and Schwann cells in ChIP-seq experiments. As indicated in Venn diagram, 94 upregulated DEG – including *Enpp2*, *Erbb3*, *Apod* or *Plp1* – and 12 downregulated DEG of our gene set have been shown in ChIP-seq to be directly regulated by SOX10 in both oligodendrocytes and Schwann cells (Fig. 3d, Supplementary Table 6).

### Confirmation of SOX10-Induced Expression of Selected Myelin-Associated Genes in JUG2 Cells

**Fig. 4:**
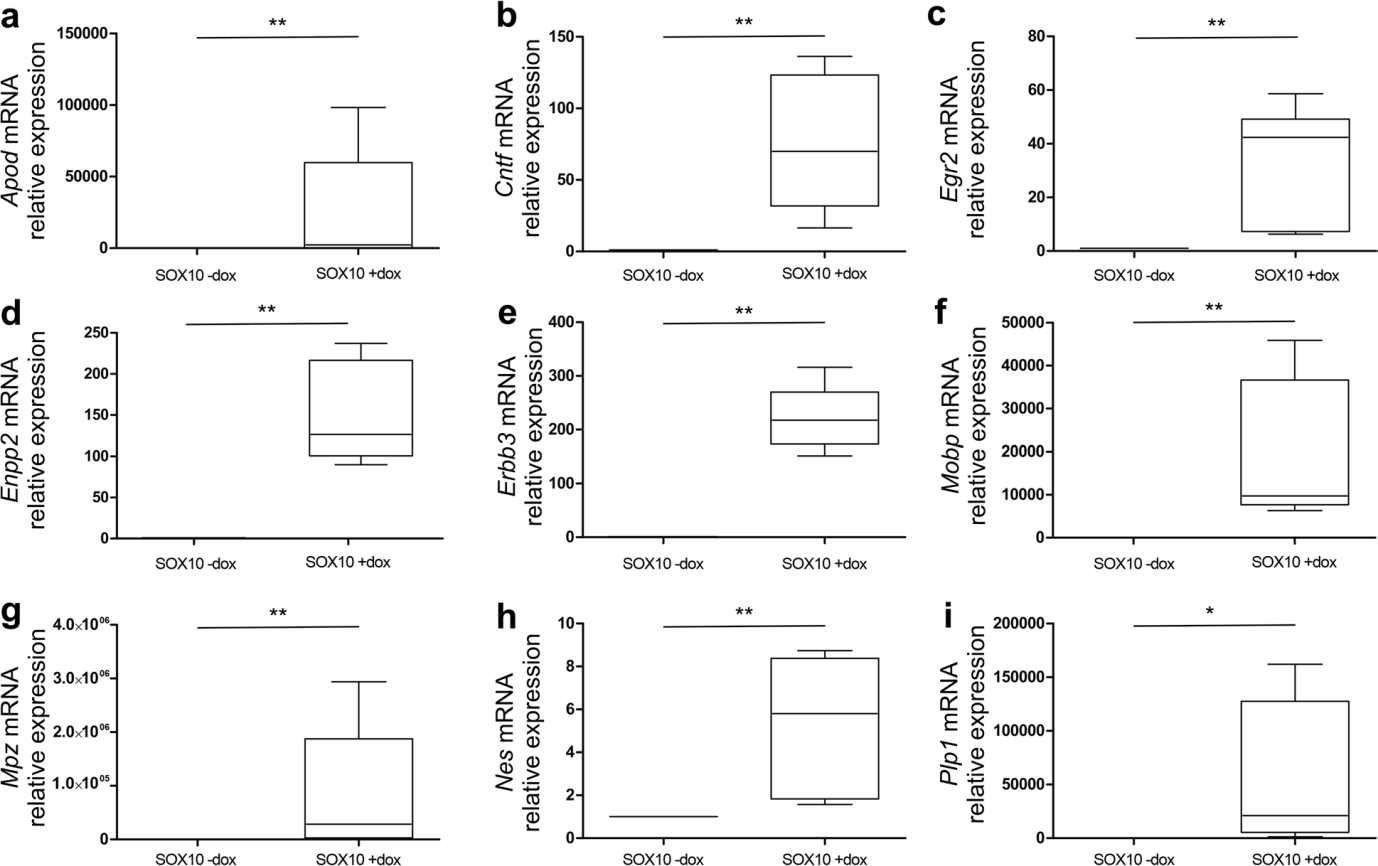
Impact of SOX10 overexpression on expression of selected genes in JUG2 cells. mRNA expression of *Apod* (**a**), *Cntf* (**b**), *Egr2* (**c**), *Enpp2* (**d**), *Erbb3* (**e**), *Mobp* (**f**), *Mpz* (**g**), *Nes* (**h**) and *Plp1* (**i**) was determined by RT-qPCR (*n* = 4–6). Data were normalized to SOX10 -dox. *p ≤ 0.05, **p ≤ 0.01 in comparison to SOX10 -dox (Mann–Whitney U test)

Induction of selected genes was confirmed using RT-qPCR. Expression of *Apod, Cntf, Egr2, Enpp2, Erbb3, Mobp, Mpz, Nes* and *Plp1* was significantly induced by SOX10 overexpression in EGC (Fig. 4a–i). In detail, expression level of all nine tested genes was markedly increased between 5- and 8.2 × 10^5^-fold compared to control (*Apod* ~ 13 000-fold, *Cntf* ~ 75-fold, *Egr2* ~ 35-fold, *Enpp2* ~ 155-fold, *Erbb3* ~ 220-fold, *Mobp* ~ 20 000-fold, *Mpz* ~ 820 000-fold, *Nes* 5-fold and *Plp1* ~ 52 000-fold).

**Fig. 5:**
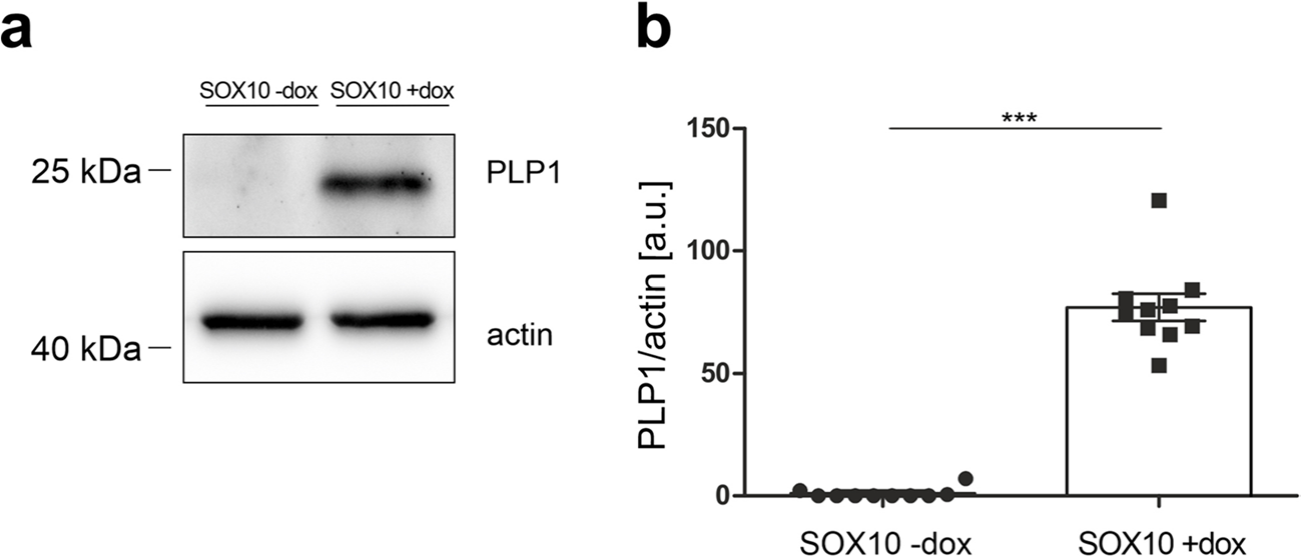
SOX10 overexpression leads to increased PLP1 levels in JUG2 cells. **a** Representative western blot of PLP1 and actin (loading control) and **b** densitometrical analysis of PLP1 expression levels. *n* = 10, ***p ≤ 0.001 (Mann–Whitney U test)

PLP1 is known as the most abundant myelin membrane protein of the CNS, but is also expressed at lower levels in the PNS as well as in the ENS. Quantification of PLP1 protein levels of SOX10 overexpressing JUG2 cells showed a significant increase of protein level by the factor of ~ 80 thereby confirming the results of microarray and RT-qPCR (Fig. 5a–b).

### Comparison of Pan-Glial SOX10 Target Genes to Human ENS-specific scRNA-Seq Dataset Highlights Novel EGC-Associated SOX10 Target Genes

In order to gain information about the relevance of the SOX10-induced DEG for the human ENS, we performed a screening for overlapping genes between SOX10-induced DEG (this study), SOX10 target genes identified in both oligodendrocytes or Schwann cells in ChIP-seq experiments (Lopez-Anido et al. 2015) and genes enriched in human single cell enteric neural clusters (Fawkner-Corbett et al. 2021).

**Fig. 6:**
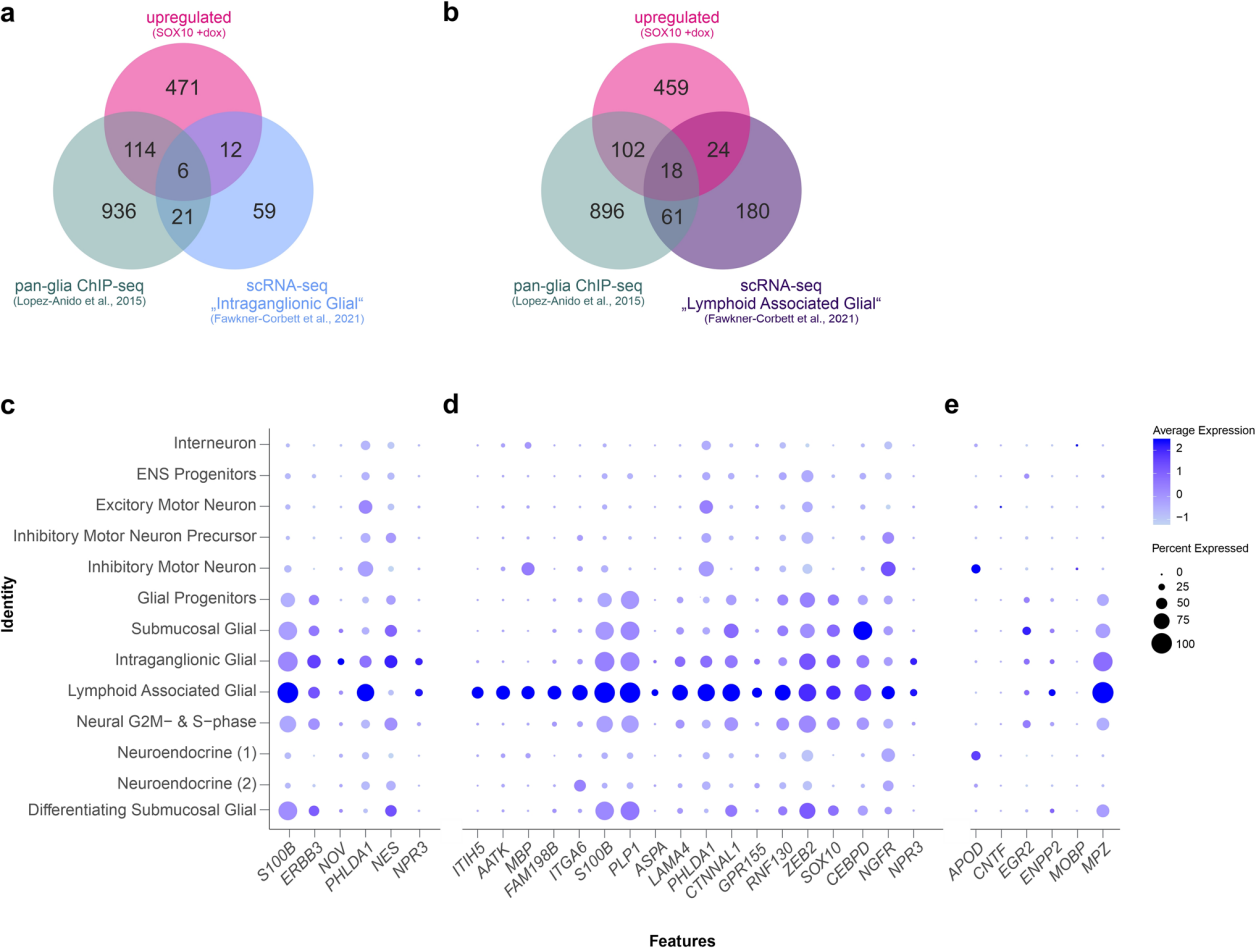
**a** Venn diagram representing the overlap between upregulated SOX10 DEG, glia-associated ChIP-seq-validated SOX10 target genes (Lopez-Anido et al. 2015), and enriched genes present in the “intraganglionic glial” cluster, as determined by scRNA-seq analysis (Fawkner-Corbett et al. 2021). **b** Venn diagram representing the overlap between upregulated SOX10 DEG, ChIP-seq validated SOX10 target genes (Lopez-Anido et al. 2015) and enriched genes present in the “lymphoid associated glial” cluster, as determined by scRNA-seq analysis (Fawkner-Corbett et al. 2021). **c**–**e** Dotplot showing the expression of overlapping SOX10 target genes of the “intraganglionic glial” cluster (c) and the “lymphoid associated” cluster (d) – as determined in (a) and (b) respectively – in cell clusters of the human ENS. Expression of selected additional SOX10 genes of interest is further shown (e). Expression data were obtained from published scRNA-seq analysis (Fawkner-Corbett et al. 2021)

Of the identified upregulated SOX10 target genes, 6 genes were significantly enriched in the “intraganglionic glial” cells cluster and 18 genes were significantly enriched in the “lymphoid associated glial” cluster (Fig. 6a–d, Supplementary Table 7).

Expression of additional SOX10 target genes of interest within the human ENS nervous system is further represented (Fig. 6e). Noteworthy, expression of *Mpz* and *Plp1* was associated with the “lymphoid associated glial” cluster. In contrast, only limited expression of *Mobp* or *Cntf* was observed in clusters corresponding to ENS cellular subtypes (Fig. 6e).

### Identification of SOX10 Target Genes Potentially Altered in Multiple Sclerosis

**Fig. 7:**
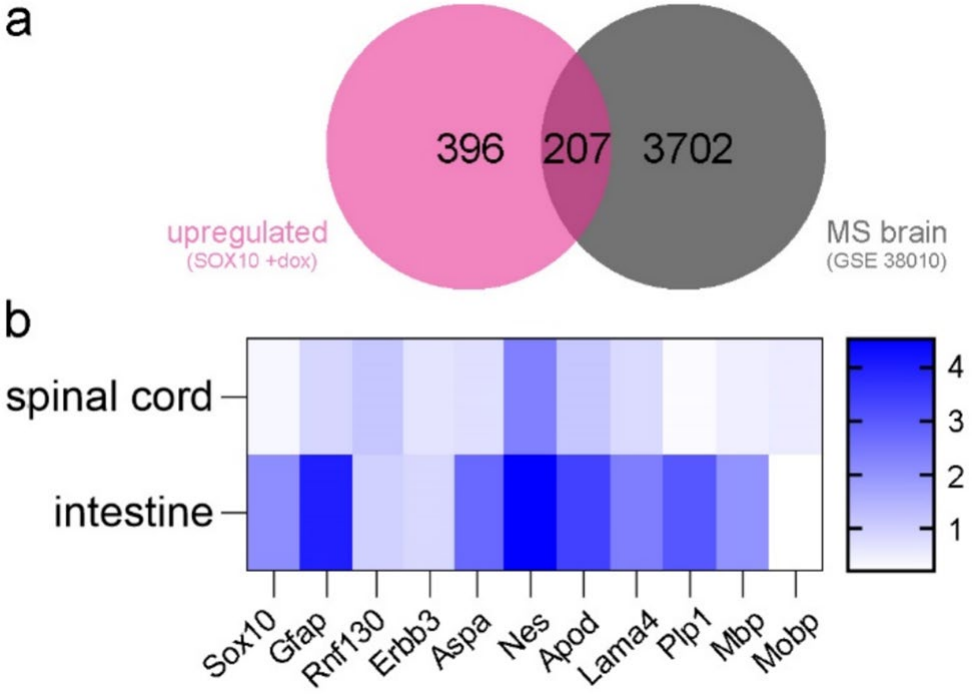
**a** Venn diagram representing the overlap between upregulated DEG induced by SOX10 overexpression in JUG2 cells and upregulated DEG in active plaques within the brain of MS patients (GSE38010)(Han et al. 2012). **b** Heatmap showing fold change in gene expression of selected SOX10 target genes in the colon tissue (*n* = 2–4 per group) and spinal cord (*n* = 4 per group) of EAE mice compared to wild-type mice as determined by RT-qPCR

In order to gain further information about the relevance of the identified SOX10 target genes in pathological processes, SOX10-induced DEG were compared to an existing transcriptomic dataset for MS patients (GSE38010). Of SOX10-induced target genes, 207 DEGs were also altered in active MS plaques in comparison to control tissue (Fig. 7a, Supplementary Table 8). Those included *S100B*, *ERBB3*, *NES*, *PLP1*, *MBP*, *AATK*, *ASPA*, *LAMA4*, *RNF130* and *PHLDA1* as DEGs associated with EGC, as well as *APOD*, *EGR2*, *ENPP2* as additional pan-glial SOX10 target genes. Expression of genes of interest, including *RNF130*, *ERBB3*, *ASPA*, *NES*, *APOD*, *LAMA4*, *PLP1*, *MBP* and *MOBP* was finally analyzed in an explorative way in the spinal cord and in intestinal tissues of EAE mice suggesting increased expression of *ASPA, NES*, *APOD*, *LAMA4*, *PLP1* and *MBP* was observed in the colon of EAE mice (Fig. 7b).

## Discussion

### Impact of Sox10 Overexpression on EGC Phenotype

In this study, we investigated the impact of SOX10 overexpression on EGC phenotype in vitro.

EGC share particular molecular regulatory networks and some specific markers including GFAP, S100B or SOX10 with peripheral Schwann cells and central glial cells (Reed et al. 2022). Nonetheless, EGC appear to have a specific molecular signature and thus differ significantly from other glial cell types (Rao et al. 2015; Progatzky and Pachnis 2022; Bubeck et al. 2023; Lefèvre et al. 2023). EGC have been shown to lose their phenotype and dedifferentiate into neural progenitors in culture (Heanue and Pachnis 2011; Guyer et al. 2023). The JUG2 cell line used in our study similarly represents a dedifferentiated state of EGC, characterized by low GFAP, S100B and SOX10 levels, but remains Nestin-positive. Interestingly, although SOX10 overexpression results in the inhibition of differentiation of enteric neural crest stem cells and enteric progenitors cells (Kim et al. 2003; Bondurand et al. 2006), JUG2 cells appear to be committed to a glial phenotype after SOX10 overexpression. Most particularly, SOX10 overexpression was associated with the induction of glial and myelin associated genes associated with both central and peripheral rather than enteric glial cells.

Among the canonical glial markers, GFAP expression has been shown to be highly variable in EGC (Boesmans et al. 2015; Cossais et al. 2019; Baidoo et al. 2023). GFAP expression in EGC is increased under inflammatory stress and neuroinflammatory disorders, including MS (Wunsch et al. 2017) and GFAP-positive EGC have recently been proposed to possess neurogenic potential (Guyer et al. 2023). In our study, we did not detect any impact of SOX10 overexpression on the expression of GFAP in our cell culture model, indicating that regulation of GFAP may rely on alternative transcriptomic pathways.

More surprisingly, whereas S100B is a known SOX10 target gene (Fujiwara et al. 2014; Lopez-Anido et al. 2015), we observed only limited effects of SOX10 overexpression on S100B expression (see also Supplementary Table 2).

### Induction of Myelin Gene Expression

A large number SOX10-induced DEG were associated with a Schwann cell phenotype, an oligodendrocyte phenotype or both. The transcription factor early growth response protein 2 (*Egr2)* is known to interact with *Sox10* during Schwann cell development (Reed et al. 2022). *Egr2* expression has not yet been related to EGC and *Egr2* appears to be expressed only at low level in the ENS, as determined from single transcriptomic data (see also Fig. 6). Ciliary neurotrophic factor (CNTF) is similarly specifically associated with Schwann cell phenotype, and might be involved in the regulation of ENS development (Chalazonitis et al. 1998; Ito et al. 2006). Amongst other genes of interest, apolipoprotein D (*APOD*) has been associated with perisynaptic Schwann cells, but its expression appears limited within the ENS (Ganfornina et al. 2010; Windster et al. 2023). The ectonucleotide pyrophosphatase/phosphodiesterase 2 (*ENPP2*), also named autotaxin (*ATX*), is another important regulator of glial physiology, both in the central and peripheral nervous system (Savaskan et al. 2007). Interestingly, *Enpp2* expression has recently been demonstrated in EGC, and may be involved in the regulation of intestinal motility (Ahmadzai et al. 2022). Myelin protein zero (*MPZ*) is a known target gene of SOX10 (Peirano et al. 2000). Within the gut, MPZ-positive cells have been described as Schwann-like enteric glia (Windster et al. 2023) or lymphoid-associated glia (Fawkner-Corbett et al. 2021). Nonetheless, immunohistological characterization of MPZ-positive cells in the human ENS suggests these cells to be extrinsic Schwann cells (Woods et al. 2020; Kapur et al. 2022). ERBB3, also known as HER3 (human epithelial growth factor receptor 3), has been shown to play important roles during ENS development and is a well characterized SOX10-target gene (Buac et al. 2008; Prasad et al. 2011). Expression of *ERBB3* has been characterized in adult human EGC (Barrenschee et al. 2015). NESTIN (*NES*) is similarly an important regulator of Schwann cell development and has been demonstrated to be expressed in adult EGC in mice (Grundmann et al. 2016).

Of the myelin-related genes induced by SOX10, proteolipid protein 1 (PLP1) is another direct SOX10-target gene, whose expression in the ENS has been well characterized in the recent years (Progatzky et al. 2021). PLP1 has been shown to be expressed in Schwann cell precursor cells and in EGC (Rao et al. 2015; Jansky et al. 2021; Kapitza et al. 2021), and its expression in intestinal tissue decreases overtime to become barely detectable in adulthood (Patyal et al. 2023). PLP1 functions in the ENS remain largely unclear, despite recent evidences indicating that PLP1-positive EGC may play a role in the regulation of intestinal motility (Rao et al. 2017; Baghdadi et al. 2022; Grubišić and Gulbransen 2022; Woods et al. 2023).

Beside the induction of peripheral glia-related genes, we also observed the aberrant induction of oligodendrocyte-specific genes, including myelin associated oligodendrocyte basic protein (*Mobp)*. As determined from single cell transcriptomic data, *Mobp* does not appear to be expressed in EGC, but is a known SOX10-target gene in oligodendrocytes. Similar SOX10-induced ectopic oligodendrocyte-associated gene expression in satellite glial cells has been observed in a mouse model (Weider et al. 2015). Furthermore, SOX10 overexpression induces the expression of oligodendrocyte-associated genes in multipotent neural precursor cells as well as in induced pluripotent stem cells (Pozniak et al. 2010; García-León et al. 2018; Neyrinck and García-León 2021).

### Identification of Novel EGC-Associated Sox10 Target Genes

Our results also shed a new light on several SOX10-target genes potentially associated with EGC phenotype and functions. Of these genes, *LAMA4*, *ZEB2*, *CTNNAL1*, *ITGA6* and *ITIH5* have previously been associated with Hirschsprung’s disease and *ZEB2* was shown to genetically interact with *SOX10* (Heanue and Pachnis 2006; Watanabe et al. 2017; Cai et al. 2018; Wang et al. 2020; Niu et al. 2020; Sun et al. 2021; Windster et al. 2023; He et al. 2023; Hou and Kang 2023). The lamina 4 gene (LAMA4) has been associated with EGC phenotype in recent scRNA-seq studies (Drokhlyansky et al. 2020).

Our results also highlight *Aatk*, *Aspa*, *Nov*/*Ccn3*, *Cebpd*, *Fam198b*, *Gpr155*, *Ngfr*, *Npr3*, *Phlda1* and *Rnf130* as potential regulators of peripheral glial phenotype (Mersmann et al. 2011; Reiprich et al. 2017; Magi et al. 2018; de la Vega Gallardo et al. 2020). *Nov* expression has been shown to be induced in differentiating enteric progenitor cells (Neckel et al. 2016). *FAM198b* has previously been associated with glial plasticity observed during perineural invasion (Zhang et al. 2019). *PHLDA1* has been reported to be involved in the regulation of Erbb, which plays a major role in ENS development (Magi et al. 2018). RNF130 has been shown to be involved in the regulation of Wnt signalling and may regulate tau protein expression (Kim et al. 2023). *GPR155*, also named LYCHOS, has recently been described as a lysosomal cholesterol sensor protein (Shin et al. 2022). Nonetheless, little is yet known about the functions of these genes of interest in the regulation of glia functions.

### Implication for Multiple Sclerosis

Expression of a large set of identified Sox10-genes target genes showed increased expression within active plaques in the CNS of MS patients (Han et al. 2012). In line with these results, induction of myelin-related genes has been confirmed in a recent study (Elkjaer et al. 2024). Besides myelin genes, *PHDLA1* has been associated with MS (Jokubaitis et al. 2023; Lerma-Martin et al. 2024). Furthermore, our data indicate that *Nes* expression is increased in the CNS of EAE mice and MS patients, confirming previous results (Snethen et al. 2008). These results also suggest that *Nes* may similarly be induced in the ENS under MS condition and that SOX10 induction may lead to the activation of genes associated with neural precursor cells in MS. Nonetheless, further studies are needed to investigate the potential role of Nestin activation in MS.

Taken together, our results support that SOX10 expression level play a key role in the regulation of both central and peripheral glial phenotype (Pingault et al. 2022). Further development of cellular tools and future studies are required to identify potential co-factors and signaling pathways involved in the fine tuning of *Sox10* expression level in EGC and in the regulation of glial phenotype both *in vitro* and *in vivo*. These steps appear as important prerequisites toward the development of a controlled stem cell-based therapy for central and peripheral neuropathy as well as for gastro-intestinal motility disorders.

## Supplementary Information

Below is the link to the electronic supplementary material.

Supplementary file1 (XLSX 920 KB)

## Data Availability

Microarray data that supports the findings of this study have been deposited in the GEO databank under the accession number GSE249205.
